# Supplementary material for: Development of a Framework for Scaling Up Community-Based Health Promotion: A Best Fit Framework Synthesis
Source: Int J Environ Res Public Health. 2022 Apr 14;19(8):4773. doi: 10.3390/ijerph19084773 (PMC9032469; doi:10.3390/ijerph19084773)
Supplement: Supplementary file 1 [file ijerph-19-04773-s001.zip › Table S1-Search strategy frameworks.pdf]

**Table S1. Search strategy: frameworks.**

| | |
|---|---|
| Behavior of interest (health innovation/ health promotion) | |
| #1 | "health innovation*" OR "health intervention*" OR "health promotion" OR "health promotion" OR "public health" OR "health program*" OR "health policy" OR "health polic*" |
| Health context (scaling up) | |
| #2 | "Scale Up" OR "scaled up" OR "Scaling up" OR "scalab*" OR "At Scale" OR "broad scale" |
| Model or theory | |
| #3 | "model*"[Title/Abstract] OR "theor*"[Title/Abstract] OR "framework*"[Title/Abstract] OR "concept*" |
| Final search | |
| #4 | #1 AND #2 AND #3 |

(*) search for all terms that begin with that word

(#) Citations in the Clipboard that are represented by the search number #, which may be used in Boolean search statements, for example, to limit the citations you have collected in the Clipboard
