# Supplementary material for: Development of a Framework for Scaling Up Community-Based Health Promotion: A Best Fit Framework Synthesis
Source: Int J Environ Res Public Health. 2022 Apr 14;19(8):4773. doi: 10.3390/ijerph19084773 (PMC9032469; doi:10.3390/ijerph19084773)
Supplement: Supplementary file 1 [file ijerph-19-04773-s001.zip › Table S2-Inclusion criteria frameworks.pdf]

**Table S2. Inclusion criteria: frameworks**

| Inclusion | Exclusion |
|---|---|
| Related to scaling up (as defined by WHO [11]) | Not related to health promotion |
| Frameworks or models, which provide a structure, by which a scaling-up process can be organized | Primary healthcare innovations and technical innovations |
| Related to physical activity or health promotion | Infectious diseases |
| | Limited to a single setting or population |
| | Languages other than English and German |

Reference:

1. World Health Organization. *Nine Steps for Developing a Scaling-Up Strategy*; World Health Organization: Geneva, Switzerland, 2010; ISBN 924150031X.
