# Supplementary material for: Development of a Framework for Scaling Up Community-Based Health Promotion: A Best Fit Framework Synthesis
Source: Int J Environ Res Public Health. 2022 Apr 14;19(8):4773. doi: 10.3390/ijerph19084773 (PMC9032469; doi:10.3390/ijerph19084773)
Supplement: Supplementary file 1 [file ijerph-19-04773-s001.zip › Table S3-Search strategy primary research studies.pdf]

Table S3. Search strategy: primary research studies

Scaling up	
#1	"Scale Up" OR "scaled up" OR "Scaling up" OR "scalab*" OR "At Scale" OR "broad scale"
Community	
#2	county OR city OR cities OR municipal* OR urban OR town OR suburb OR rural OR "local authority" OR communit* OR community-based OR neighborhood OR neighbourhood
Health promotion/ physical activity	
#3	"health promotion" OR "health intervention*" OR "health program*" OR "physical activit*" OR "physical inactivit*" OR exercise OR "exercise" OR "physical fitness" OR sport* OR bike OR bicycle* OR bicycling OR bicycling OR walking OR "walking" OR "active transport" OR sedentary OR "active living" OR "leisure activit*" OR obes* OR "obesity" OR overweight OR "weight loss"
Final search	
#4	#1 AND #2 AND #3

(*) search for all terms that begin with that word

(#) Citations in the Clipboard that are represented by the search number #, which may be used in Boolean search statements, for example, to limit the citations you have collected in the Clipboard
