# Supplementary material for: Development of a Framework for Scaling Up Community-Based Health Promotion: A Best Fit Framework Synthesis
Source: Int J Environ Res Public Health. 2022 Apr 14;19(8):4773. doi: 10.3390/ijerph19084773 (PMC9032469; doi:10.3390/ijerph19084773)
Supplement: Supplementary file 1 [file ijerph-19-04773-s001.zip › Table S4-Inclusion criteria primary research studies.pdf]

**Table S4. Inclusion criteria: primary research studies**

| Inclusion | Exclusion |
|---|---|
| Primary research study | Dissemination only |
| Reporting of information on the scaling-up process | Primary healthcare innovations and technical innovations |
| Related to physical activity and health promotion | Infectious diseases |
| | Limited to health-related results, no results of the scaling-up process |
| | Languages other than English and German |
